# A real-world study of cardiac events in > 3700 patients with HER2-positive early breast cancer treated with trastuzumab: final analysis of the OHERA study

**DOI:** 10.1007/s10549-018-5058-6

**Published:** 2018-11-30

**Authors:** Elisabet Lidbrink, E. Chmielowska, B. Otremba, A. Bouhlel, S. Lauer, M. Liste Hermoso, E. Nüesch, M. Shing, V. Misra

**Affiliations:** 1 Karolinska Institute, Karolinska University Hospital, Stockholm, Sweden; 2 Regionalne Centrum Onkologii, Medical University Nicolaus Copernicus, Bydgoszcz, Toruń, Poland; 3 Onkologische Praxis Oldenburg, Oldenburg, Germany; 4 Global Pharma Development, F. Hoffmann-La Roche Ltd, Basel, Switzerland; 5 Biostatistics, F. Hoffmann-La Roche Ltd, Basel, Switzerland; 6 Genentech, Inc., South San Francisco, CA USA; 7 The Christie, Manchester, UK

**Keywords:** Trastuzumab, Cardiac adverse events, HER2-positive early breast cancer, HER2-targeted therapies, Real-world population

## Abstract

**Purpose:**

Cardiac dysfunction risk associated with intravenous trastuzumab (H IV) treatment may differ in real-world practice versus randomized trials. We investigated cardiac events in patients with HER2-positive early breast cancer (EBC) treated with H IV as adjuvant therapy in routine practice.

**Methods:**

The observational study of cardiac events in patients with HER2-positive EBC treated with Herceptin (OHERA; NCT01152606) enrolled patients with stage I–IIIb disease eligible for H IV in the adjuvant setting per the European Summary of Product Characteristics (SmPC). Primary outcomes were symptomatic congestive heart failure incidence (CHF; New York Heart Association class II–IV) and cardiac death. Patient visits/assessments were per local practice.

**Results:**

3733 Patients received ≥ 1 H IV dose per local practice; 88.9% received H IV for > 300 days (median follow-up: ~ 5 years). Prior to disease recurrence (if any), symptomatic CHF occurred in 106 patients (2.8%); 6 (0.2%) cardiac deaths occurred (5 in patients with cardiac disease history). Median time to symptomatic CHF onset was 5.7 months (95% CI 5.3–6.5); 77/106 (72.6%) patients with symptomatic CHF achieved resolution. CHF incidence was higher in patients ≥ 65 years, and those with pre-existing cardiac conditions, hypertension, or left ventricular ejection fraction ≤ 55% at baseline.

**Conclusions:**

OHERA is the largest prospective observational study to investigate the cardiac safety of H IV as adjuvant EBC therapy in a real-world setting. Symptomatic CHF and cardiac event incidences were consistent with randomized trials in this setting and baseline risk factors identified in the H IV European SmPC.

**Electronic supplementary material:**

The online version of this article (10.1007/s10549-018-5058-6) contains supplementary material, which is available to authorized users.

## Introduction

As of September 2018, > 2.7 million patients with breast cancer had received intravenous or subcutaneous trastuzumab (Herceptin^®^, F. Hoffmann-La Roche Ltd., Basel, Switzerland; intravenous trastuzumab (H IV) and subcutaneous trastuzumab (H SC), respectively) in clinical trial or real-world settings (Roche internal data).

The phase 3 HERceptin Adjuvant [HERA; Breast International Group (BIG) 01-01], National Surgical Adjuvant Breast and Bowel Project (NSABP)-B31/N9831, and Breast Cancer International Research Group (BCIRG)-006 studies demonstrated the clinical benefits of 1 year of H IV as adjuvant therapy for the treatment of patients with HER2-positive early breast cancer (EBC) [1–3]. H IV was approved for the treatment of HER2-positive EBC in the adjuvant setting in Europe, and many other countries worldwide, on the basis of significant improvements in disease-free survival (DFS) after 1 year, per the primary analysis of the HERA study (unadjusted hazard ratio for DFS 0.54 [95% confidence interval (CI) 0.44–0.67]; *P* < 0.0001) [1, 4]. Subsequent analyses of HERA, based on median follow-up periods of up to 11 years, reinforced the DFS benefits associated with 1 year of H IV treatment [1, 5–7] and overall survival was shown to be prolonged in patients who received H IV, compared with control arms, in long-term analyses from each of the three H IV adjuvant EBC studies [3, 5, 7, 8].

Anti-HER2 treatment is associated with increased risk of cardiac dysfunction, and particularly congestive heart failure (CHF) [1, 3, 4]. Risk of cardiac failure in H IV-treated patients is relevant in real-world clinical practice and may differ from the risk observed in a randomized, controlled clinical trial setting with strict entry criteria and follow-up procedures. Despite the increased risk of cardiac dysfunction associated with H IV in patients with breast cancer, the incidence of symptomatic CHF and significant decreases in left ventricular ejection fraction (LVEF) were low (1.7 and 7.1%, respectively) in the HERA study at 1-year median follow-up, and remained low during subsequent longer-term follow-up analyses (1.9 and 9.8%, respectively after a median follow-up of 3.6 years) [1, 9]. Moreover, long-term analyses of adjuvant EBC trials have suggested the majority of cardiac events are reversible [10, 11].

The present observational study of cardiac events in patients with HER2-positive EBC treated with Herceptin (OHERA; NCT01152606) was conducted as a post-authorization commitment following European Union (EU) approval of H IV in the adjuvant EBC setting. OHERA is a non-interventional, single-cohort safety study designed to investigate the incidence of cardiac events in a real-world population of patients with HER2-positive EBC being treated with H IV in the adjuvant setting as per the EU Summary of Product Characteristics (SmPC) [4], in routine clinical practice.

Here we report the final analysis of the OHERA study. The primary objective of the OHERA study was to observe, in the routine clinical practice setting, the incidence of symptomatic CHF and cardiac death in patients with HER2-positive EBC who received H IV per the approved EU SmPC. Secondary objectives were to explore potential risk factors for symptomatic CHF and cardiac death, to observe the time to onset and resolution of symptomatic CHF and other significant cardiac conditions, and to record the incidence of asymptomatic left ventricular dysfunction.

## Methods

### Study design

Patients with HER2-positive EBC (stage I–IIIb) being considered for treatment with H IV in the adjuvant setting per the EU SmPC were enrolled, treated, and monitored according to local practice [4]. No additional procedures/patient visits outside routine clinical practice were performed during the study. The study was conducted in accordance with the Declaration of Helsinki and the laws and regulations of each country in which the study was performed. The study protocol was approved by the Independent Ethics Committee or Institutional Review Board for each site prior to starting the study. Eligible patients were required to provide written informed consent prior to enrollment.

### Patients

Due to the non-interventional nature of the study, no specific inclusion or exclusion criteria beyond the indication and contraindications in the H IV EU SmPC were applied when enrolling patients [4]. Patients with HER2-positive EBC whose tumors had either HER2 overexpression or *HER2* gene amplification as determined by an accurate and validated assay were considered to be eligible for enrollment and treatment with H IV per the EU SmPC [4]. Patients with contraindications to H IV per the EU SmPC, i.e., those with known hypersensitivity to H IV, murine proteins, or any of the excipients, and patients with severe dyspnea at rest due to complications of advanced malignancy, or requiring supplementary oxygen therapy [4], were not eligible for this study.

Consenting patients were enrolled into the study sequentially without any pre-selection process, and irrespective of whether they subsequently received H IV following physician assessment of the benefit:risk balance.

Treatment with H IV was administered per local practice and in accordance with the EU SmPC. At study initiation in 2007, the approved treatment regimen in the EU SmPC for H IV as adjuvant therapy for HER2-positive EBC was monotherapy following surgery, chemotherapy, and/or radiotherapy based on the phase 3 HERA study results; subsequently in 2011, during the course of the OHERA study, approved treatment regimens in the EU SmPC were updated to include concurrent use of H IV with chemotherapy in the adjuvant treatment of patients with HER2-positive EBC as part of a treatment regimen consisting of doxorubicin and cyclophosphamide followed by H IV plus paclitaxel (PTX) or docetaxel, or H IV plus docetaxel plus carboplatin [4].

### Outcomes

The primary safety outcomes were incidence of symptomatic CHF [New York Heart Association (NYHA) class II–IV] and incidence of cardiac death. Secondary outcomes included time to onset and time to recovery of symptomatic CHF and other cardiac conditions, and incidence of asymptomatic left ventricular dysfunction (evaluated as the incidence of significant LVEF drop; defined as at least one drop in LVEF of ≥ 10 percentage points from baseline to < 50%). Exploratory analyses of potential risk factors associated with incidence of symptomatic CHF were also conducted.

### Assessments

Cardiac assessments were to be performed per local practice and/or in accordance with guidance contained in the approved H IV EU SmPC, which recommends LVEF assessments be performed at the time of treatment initiation (baseline), every 3 months during treatment, and at 6, 12, and 24 months after treatment discontinuation (exact schedules varied according to local practice). The frequency of LVEF assessments could be increased if clinically indicated, e.g., in cases of LVEF drop or presence of symptoms of cardiac dysfunction. Cardiac function and LVEF were monitored by echocardiogram (ECHO), multiple-gated acquisition (MUGA) scan, magnetic resonance imaging, or other methods per local practice. Data were collected on baseline demographics, breast cancer disease characteristics, medical history and pre-existing conditions ongoing at baseline (focusing on cardiac-related conditions and potential risk factors for cardiac events such as hypertension, diabetes mellitus, obesity, smoking habits, ischemic heart disease, or arrhythmias), previous anticancer therapy (and concurrent treatments if still ongoing at the time of starting H IV), hormonal therapy, radiotherapy treatment, and disease recurrence.

Patient data were to be collected for up to 5 years after the first H IV administration, or until death, loss to follow-up or withdrawal of consent, whichever occurred first.

### Statistical analysis

As this study was not hypothesis testing, no formal sample size calculation was performed and all analyses are descriptive. However, a target enrollment of approximately 3800 patients treated with H IV was agreed with the European health authorities and considered sufficient to detect a 1% increase in CHF based on the background incidence of up to 4%, as reported in previous adjuvant EBC trials [2]. With the target enrollment of 3800 patients, the width of the Pearson–Clopper 95% CI remained reasonably small when assuming incidence rates ranging from 2% (95% CI 1.58–2.50) to 5% (95% CI 4.33–5.74).

The final analysis was conducted in the safety population, defined as all enrolled patients with EBC (stage I–IIIb) who received at least one dose of H IV in the adjuvant setting.

Demographic and baseline characteristic data were summarized for all patients in the safety population. CHF and cardiac death were summarized as incidence with two-sided Pearson–Clopper 95% CI. Only cardiac events and LVEF assessments prior to disease recurrence were included in the analyses. Time to event onset and resolution were analyzed using Kaplan–Meier methodology.

## Results

**Fig. 1:**
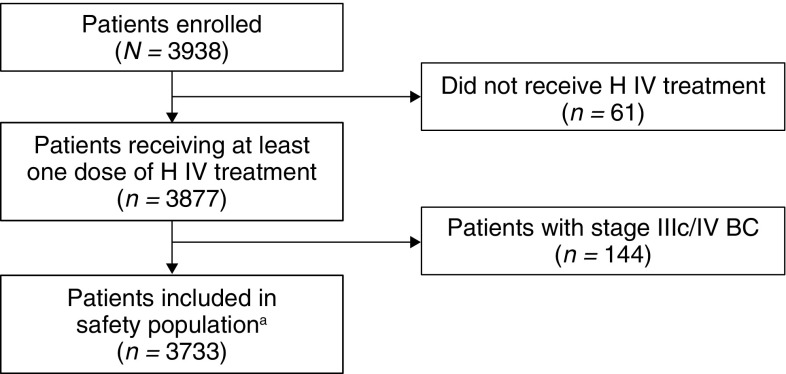
Patient disposition. *BC* breast cancer, *EBC* early breast cancer, *H IV* intravenous trastuzumab. ^a^Patients with stage I–IIIb EBC at study entry

In total, 3938 patients were enrolled between August 2007 and November 2010 at 199 sites across Austria, Belgium, Germany, Hungary, Italy, Poland, Spain, Sweden, and the UK. Of these, 3733 (95%) individuals had stage I–IIIb breast cancer and received at least one dose of study treatment, and therefore comprised the safety population (Fig. 1). The median age of patients in the safety population was 55.0 years (range 21–86 years; Table 1) and > 99% of patients were women. The majority of patients (> 80%) had stage I or II breast cancer, 62.6% were estrogen receptor-positive, and 90.2% had received prior anthracycline-based chemotherapy before starting H IV treatment (Table 1). Concurrent medications are provided in Supplementary Table 1. The median LVEF at baseline was 64.0 (range 36–90) and 356 (9.5%) patients had a family history of CHF. Overall, 1574 (42.2%) patients had ≥ 1 pre-existing cardiovascular medical condition representing a risk for symptomatic CHF including hypertension (28.3%), thyroid gland disorder (10.4%), dyslipidemia (7.5%), and diabetes mellitus (4.7%) (Table 1); 1143 patients (30.6%) were current or ex-smokers.

**Table 1:** Baseline characteristics (safety population)

Characteristics	All patients (*N* = 3733)
Median age (range), years	55.0 (21–86)
BMI ≥ 25 kg/m^2^, *n* (%)	1940 (52.0)
Current or ex-smoker, *n* (%)	1143 (30.6)
Disease characteristics, *n* (%)	
Tumor status	
Stage I	1257 (33.7)
Stage II	1836 (49.2)
Stage IIIa	521 (14.0)
Stage IIIb	115 (3.1)
Unknown	4 (0.1)
Positive lymph node status	1666 (44.6)
Estrogen receptor status	
Positive	2336 (62.6)
Negative	1392 (37.3)
Undetermined	5 (0.1)
Prior baseline treatments for breast cancer, *n* (%)^a^	
Anthracyclines	3369 (90.2)
Taxanes	1260 (33.8)
Radiotherapy	1337 (35.8)
Left-side radiotherapy	861 (23.1)
Hormonal therapy^b^	55 (1.5)
Cardiac status at study entry	
Median LVEF at baseline, % (range)	64.0 (36–90)^c^
Diagnosis of active cardiac failure at baseline, *n* (%)	
NYHA class I	37 (1.0)
NYHA class II	11 (0.3)
NYHA class III/IV	0
History of cardiac failure, *n* (%)	49 (1.3)
Family history of CHF, *n* (%)	356 (9.5)
Pre-existing medical conditions representing a risk of CHF, *n* (%)^d^	
Hypertension	1057 (28.3)
Thyroid gland disorder	388 (10.4)
Dyslipidemia	279 (7.5)
Diabetes mellitus	177 (4.7)

*BMI* body mass index, *CHF* congestive heart failure, *LVEF* left ventricular ejection fraction, *NYHA* New York Heart Association

^a^Treatments given concurrently with H IV are not included here

^b^Including antiestrogens, aromatase inhibitors, gonadotrophin and analogs, and sex hormones

^c^Based on 3425 patients in the safety population with a numerical baseline LVEF value available

^d^Pre-existing medical conditions representing a risk of CHF reported in > 4% of the safety population

Overall, 799 (21%) patients withdrew from the study prematurely. Reasons for premature withdrawal included death (*n* = 263), loss to follow-up (*n* = 373), withdrawal of consent (*n* = 55), or admin/other reasons (*n* = 108).

The median duration of follow-up was approximately 5.0 years (1829 days, interquartile range 1793–1867 days).

### H IV treatment characteristics

The median duration of H IV adjuvant EBC treatment in the safety population was 11.8 months, and 88.9% (*n* = 3318) of patients were treated for > 300 days. A small number of patients (78 [2.1%]) had a total treatment duration of > 420 days, most likely due to treatment delays or interruptions.

Patients who experienced symptomatic CHF during the study had shorter median treatment duration and reduced treatment exposure as compared with those who did not have CHF during the study (median treatment duration 6.4 vs. 11.8 months; median cumulative dose 4117 vs 7080 mg, respectively).

### Incidence of symptomatic CHF and cardiac death

Symptomatic CHF (NYHA class II–IV) was reported in 106 (2.8%) patients, 91.5% (97/106) of whom received at least one treatment for CHF. Resolution of symptomatic CHF was achieved in 72.6% (77/106) of these individuals (as per the treating physician’s assessment) (Table 2). The most common treatments prescribed for the treatment of CHF included β-adrenoreceptor-blocking agents, angiotensin-converting enzyme inhibitors, and diuretics.

**Table 2:** Cardiac safety: incidence and resolution of CHF, and significant LVEF drops

Outcome	All patients (*N* = 3733)
Incidence of symptomatic CHF, *n* (%) (95% CI)	106 (2.8) (2.3–3.4)
Patients with CHF who achieved resolution, *n*/*N* (%)	77/106 (72.6)
Incidence of severe CHF (NYHA class III/IV), *n* (%)	38 (1.0)
Incidence of cardiac death, *n* (%) (95% CI)	6 (0.2) (0.1–0.4)
Incidence of significant LVEF drop, *n* (%)^a^	251 (7.6)
Patients with significant LVEF drop who achieved resolution, *n*/*N* (%)^b^	169/251 (67.3)

*CHF* congestive heart failure, *CI* confidence interval, *LVEF* left ventricular ejection fraction, *NYHA* New York Heart Association

^a^Evaluated in patients treated with H IV who had one numerical baseline LVEF value, and at least one numerical post-baseline LVEF value (*N* = 3291)

^b^Resolution of LVEF defined as at least one LVEF value ≥ 50% after a significant LVEF drop

**Fig. 2:**
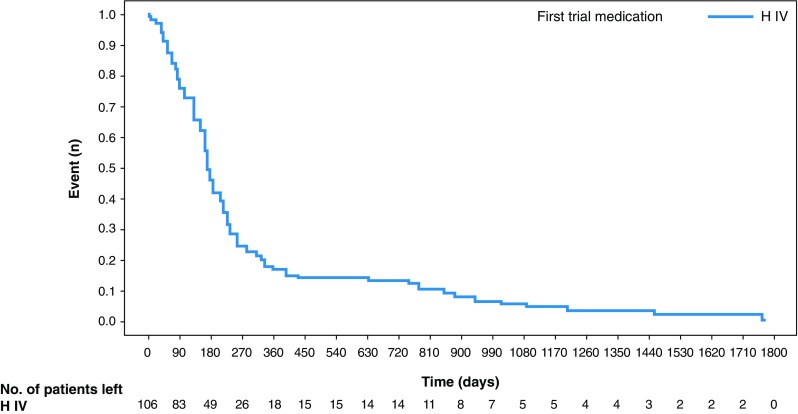
Kaplan–Meier curves for the time to onset of the first symptomatic CHF (in patients with CHF event). *CHF* congestive heart failure, *H IV* intravenous trastuzumab

The median time to onset of symptomatic CHF was 5.7 months (95% CI 5.3–6.5; Fig. 2) and the median time from onset to resolution was 9.9 months (95% CI 8.3–13.6).

Severe CHF (NYHA class III or IV) occurred in 38 (1.0%) patients and cardiac death occurred in 6 (0.2%) (Table 2). Five of the six patients who experienced cardiac death had a previous history of cardiovascular disease. The median time to cardiac death was 4.3 years (range 0.2–4.8).

In total, 264 patients died during the full study period (including deaths that occurred after disease recurrence). Of the deaths reported, 205 were related to metastases or disease progression, 14 were attributed to other cancers (cervical, ovarian, bladder, pancreatic, gastrointestinal, leukemia, glioblastoma, renal cancer, and neoplasm), and 11 were cardiac deaths.

### Incidence and resolution of significant LVEF drop

A significant drop in LVEF was observed in 251/3291 patients (7.6%), among whom resolution was documented in 169 (67.3%) (Table 2). The median time to onset of significant LVEF drop was 6.6 months (95% CI 5.8–7.2); and the median time from onset to resolution of LVEF drop was 8.8 months (95% CI 5.7–13.6).

The median LVEF was approximately 60% at baseline in patients who experienced symptomatic CHF. In these patients, LVEF generally decreased during the first 3 months after initiation of H IV, was lowest during Months 4–9, before stabilizing above 50% during Months 13–18 (Supplementary Fig. 1).

### Additional cardiac adverse events

In total, 652 (17.5%) patients experienced cardiac adverse events (AEs) prior to disease recurrence that were not classified as symptomatic CHF (individual events summarized in Table 3). When considering only the highest-grade AE experienced by an individual patient, most non-CHF cardiac AEs were Grade 1 (7.5%, 280 patients) or Grade 2 (7.1%, 264 patients). The overall incidence of Grade 3 or higher non-CHF cardiac events was low; incidence of Grade 3, 4, and 5 events was 2.4% (90 patients); 0.3% (11 patients), and 0.2% (6 patients), respectively.

**Table 3:** Incidence and severity of cardiac events not classified as symptomatic CHF

Cardiac events not classified as symptomatic CHF, *n* (%)	Any grade	NCI-CTCAE Grade 1	NCI-CTCAE Grade 2	NCI-CTCAE Grade 3	NCI-CTCAE Grade 4	NCI-CTCAE Grade 5
Acute myocardial infarction	15 (0.4)	0	3 (< 0.1)	6 (0.2)	5 (0.1)	1 (< 0.1)
Severe arrhythmia	103 (2.8)	49 (1.3)	38 (1.0)	15 (0.4)	1 (< 0.1)	0
Hypertension^a^	129 (3.5)	40 (1.1)	68 (1.8)	19 (0.5)	0	0
Angina (stable or unstable)	16 (0.4)	6 (0.2)	7 (0.2)	3 (< 0.1)	0	0
Ischemic heart disease	22 (0.6)	12 (0.3)	6 (0.2)	3 (< 0.1)	0	1 (< 0.1)
Valvular dysfunction	148 (4.0)	115 (3.1)	22 (0.6)	5 (0.1)	5 (0.1)	1 (< 0.1)
Peripheral ischemic disease	2 (< 0.1)	1 (< 0.1)	0	1 (< 0.1)	0	0
Cerebrovascular ischemia/hemorrhage	7 (0.2)	1 (< 0.1)	3 (< 0.1)	2 (< 0.1)	1 (< 0.1)	0
Sudden death	1 (< 0.1)	0	0	0	0	1 (< 0.1)
Other^b^	347 (9.3)	129 (3.5)	166 (4.4)	48 (1.3)	2 (< 0.1)	2 (< 0.1)

Data are number of events (%); denominator for percentage calculations is the safety population (*N* = 3733). Includes only the highest-grade adverse event per patient per event

*CHF* congestive heart failure, *NCI-CTCAE* National Cancer Institute-Common Terminology Criteria for Adverse Events

^a^AE grade missing for two patients

^b^Other cardiac events were described by open text

### Baseline risk factors associated with increased incidence of CHF

**Fig. 3:**
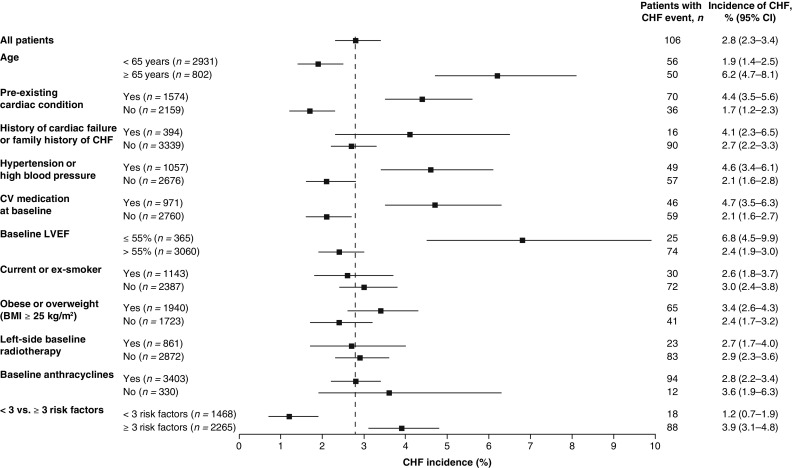
Incidence of CHF according to potential risk factors present at baseline. *BMI* body mass index, *CHF* congestive heart failure, *CI* confidence interval, *CV* cardiovascular, *LVEF* left ventricular ejection fraction. Vertical dotted line represents the incidence of CHF in the overall study population

The incidence of CHF was higher in patients with the following baseline characteristics: pre-existing cardiac conditions, use of cardiovascular medications, hypertension, LVEF ≤ 55%, body mass index (BMI) ≥ 25 kg/m^2^, age ≥ 65 years, family history of CHF or history of cardiac failure at study entry, and history of CHF or diagnosis of CHF active at study entry (Fig. 3). The incidence of CHF was not higher in patients who had received prior anthracycline therapy at baseline or in those who had received prior left-side radiotherapy. Although the incidence of CHF in OHERA was increased in patients with LVEF ≤ 55%, the incidence of severe CHF in the overall population was consistent with the incidence observed in the HERA study, in which patients were required to have an LVEF ≥ 55% [1, 6, 9].

## Discussion

The incidence rates of symptomatic CHF and cardiac death in the real-world population in the OHERA study were low and consistent with cardiac results from the HERA phase 3 randomized clinical trial (Supplementary Table 2) [1, 6, 7, 9]. Incidence of severe CHF in OHERA was also comparable with reports from the HERA study (1.0 vs. 0.5–0.8% [1], respectively) [1, 6, 9] and slightly lower than the incidence reported in most other adjuvant EBC trials (Supplementary Table 2) [3, 8, 12, 13]. The majority of patients treated per routine clinical practice in OHERA had an overall H IV treatment duration of approximately 1 year, consistent with the recommended treatment duration for EBC patients as described in the current H IV EU SmPC [4].

For most patients who experienced symptomatic CHF or significant LVEF drop in OHERA, the events began during treatment and resolved during the study period, suggesting that the cardiotoxicity associated with trastuzumab may be reversible, and that standard cardiac medications may lead to resolution of these cardiac events. This is consistent with data from HERA, which showed that the majority of patients recovered from non-fatal cardiac events including confirmed LVEF drop (recovery rate 83.3%), symptomatic CHF (recovery rate 78.1%), and severe CHF (recovery rate 69.2%) [9]. This is also consistent with the cardiac safety analyses of the other H IV adjuvant EBC trials, in which the majority of patients with cardiac events were recorded as having improved cardiac function during long-term follow-up analyses [3, 10, 11, 13, 14].

Importantly, patients were enrolled in OHERA according to the approved H IV EU SmPC and local practice, and approximately 10% of the patients in OHERA had an LVEF ≤ 55% at baseline; whereas, the HERA study enrolled only women with a baseline LVEF of ≥ 55% after completing chemotherapy or radiotherapy. Nevertheless, cardiac events in OHERA were consistent with that observed in the HERA study. Differences observed in the incidence of significant LVEF drops in OHERA (7.6%) compared to HERA (from 3.6% confirmed to 9.8% unconfirmed) [9], and as reported in other H IV EBC studies (Supplementary Table 2) are likely influenced by different study definitions for ‘significant’ LVEF drops and different follow-up periods.

Previous observational studies have reported rates of H IV-related cardiotoxicity (including both asymptomatic and symptomatic cardiac events) ranging from 15 to 27% [15–17]. Additionally, retrospective studies conducted in Israel and the US have reported cardiotoxicity/cardiac event rates ranging from 21 to 29% [18–20]. A recent population-based cohort study, including 4082 H IV-treated patients with breast cancer and a median age between 52 and 58 years [21], depending on the chemotherapy partner received reported higher cumulative incidence rates of cardiotoxicity following trastuzumab therapy than have been reported in previous clinical trials. These differences reflect variation in the definition of endpoints, duration of follow-up, and the characteristics of patients included in these analyses, all of which make cross-study comparisons challenging both between real-world studies and with randomized controlled trials. Real-world analyses conducted in restricted geographic regions are also likely to be influenced by regional differences in clinical practice, such as the use of radiotherapy and anthracyclines (although it should be noted that over 90% of patients in OHERA had received prior anthracycline therapy) [16, 20]. The prevalence of recognized risk factors for cardiotoxicity in different study populations is also important. For example, the increased rates of cardiac events reported in two large retrospective studies in the US may at least partially relate to those studies focusing on elderly patients (mean age 76 years [19] and median age 71 years in the overall population [including patients not treated with H IV]) [18], vs. median age 55 years in OHERA, a demographic group commonly underrepresented in clinical trials and also recognized as at increased risk for cardiotoxicity [4, 18, 19]. In addition, most cardiac events are reported to be reversible in observational studies and long-term follow-up analyses of the H IV adjuvant EBC trials [6, 11, 15–17].

The incidence of CHF in OHERA was low overall, but higher in selected subgroups including patients with recognized cardiac conditions at baseline, older patients (< 65 vs. ≥ 65 years), and patients with lower baseline LVEF (≤ 55% vs. > 55%). These observations are consistent with the results of NSABP-31, which showed that the relative risk of CHF increases significantly with age and with impairment in LVEF either at baseline or at the end of chemotherapy (doxorubicin plus cyclophosphamide) [13]. In particular, data from NSABP-31 showed that the risk of CHF increased significantly in patients aged 50–59 and ≥ 60 years relative to those aged < 50 years, and in patient subgroups with a baseline LVEF of 50–54% versus those with an LVEF of 55–64 and ≥ 65% [13]. Thus, relatively small differences in age or baseline LVEF may be associated with increases in the risk of CHF and may explain differences in CHF incidence between studies. Interestingly, the incidence of CHF was not higher in patients who had received left-side radiotherapy at baseline in OHERA, compared with those who did not.

The potential impact of treatment regimens comprising H IV plus anthracyclines on cardiotoxicity rates was demonstrated in a Chinese observational study, which reported that patients with EBC who received H IV plus anthracyclines, had a higher rate of cardiotoxicity than that observed in patients who received H IV without anthracyclines [16]. This association of cardiotoxicity between H IV and patients who received baseline anthracyclines was not seen in OHERA, and may have been due to clinical factors that allowed patients to receive baseline anthracyclines.

In summary, our data support previous reports that the majority of cardiac events are asymptomatic or mild and that the rates of severe or refractory CHF in patients treated with H IV are low in real-world populations [16, 17, 22].

This study has potential limitations, in addition to those intrinsic to its observational, non-interventional nature. Since patients were selected for treatment with H IV in accordance with the EU SmPC, this may have resulted in the selection of patients with different baseline characteristics compared with those in previous trastuzumab randomized clinical trials in EBC. Subjective assessment of the risk of cardiotoxicity associated with H IV by the patient or physician may have led to inadvertent selection of patients according to prognosis (channeling bias) [23]. Due to the non-interventional nature of the study, there was increased potential for patients to miss an LVEF assessment. Resolution of symptomatic CHF was defined by the treating physician and the exact definition of resolution could have differed between physicians. Importantly, recruitment of patients in OHERA was guided by indications in the EU SmPC at the time the study was initiated. Thus, although this study provides strong data on cardiac risks in a patient population treated with H IV as adjuvant EBC therapy in routine clinical practice, the study population differs from those in previous randomized controlled trials and observational studies, limiting the direct comparability of our results.

In conclusion, OHERA is the largest prospective observational study (> 3700 treated patients) to date to investigate the cardiac safety of H IV as adjuvant EBC therapy in a real-world patient population. The study’s final results are consistent with cardiac safety data reported in previous clinical trials that evaluated H IV treatment in the adjuvant setting for HER2-positive EBC [1, 2, 5–7, 9], such as the HERA study [1], and are consistent with the baseline risk factors for CHF as reported in the EU H IV SmPC [4].

## Electronic supplementary material

Below is the link to the electronic supplementary material.

Supplementary material 1 (DOCX 120 KB)

## Data Availability

Qualified researchers may request access to individual patient-level data through the clinical study data request platform (http://www.clinicalstudydatarequest.com). Further details on Roche's criteria for eligible studies are available here (https://clinicalstudydatarequest.com/Study-Sponsors/Study-Sponsors-Roche.aspx). For further details on Roche's Global Policy on the Sharing of Clinical Information and how to request access to related clinical study documents, see here (https://www.roche.com/research_and_development/who_we_are_how_we_work/clinical_trials/our_commitment_to_data_sharing.htm).

## Abbreviations

AC-T: Doxorubicin and cyclophosphamide followed by docetaxel
AE: Adverse event
BCIRG: Breast Cancer International Research Group
BIG: Breast International Group
BMI: Body mass index
CHF: Congestive heart failure
CI: Confidence interval
DFS: Disease-free survival
EBC: Early breast cancer
ECHO: Echocardiogram
H IV: Intravenous trastuzumab
H SC: Subcutaneous trastuzumab
LVEF: Left ventricular ejection fraction
MUGA: Multiple-gated acquisition
NCI-CTCAE: National Cancer Institute-Common Terminology Criteria for Adverse Events
NSABP: National Surgical Adjuvant Breast and Bowel Project
NYHA: New York Heart Association
PTX: Paclitaxel
SmPC: Summary of Product Characteristics
