# Supplementary material for: A real-world study of cardiac events in > 3700 patients with HER2-positive early breast cancer treated with trastuzumab: final analysis of the OHERA study
Source: Breast Cancer Res Treat. 2018 Nov 30;174(1):187–96. doi: 10.1007/s10549-018-5058-6 (PMC6418299; doi:10.1007/s10549-018-5058-6)
Supplement: Supplementary file 1 — Supplementary material 1 (DOCX 120 KB) [file 10549_2018_5058_MOESM1_ESM.docx]

**Electronic supplementary material**

**Supplementary Figure 1** LVEF assessments over time in patients with CHF

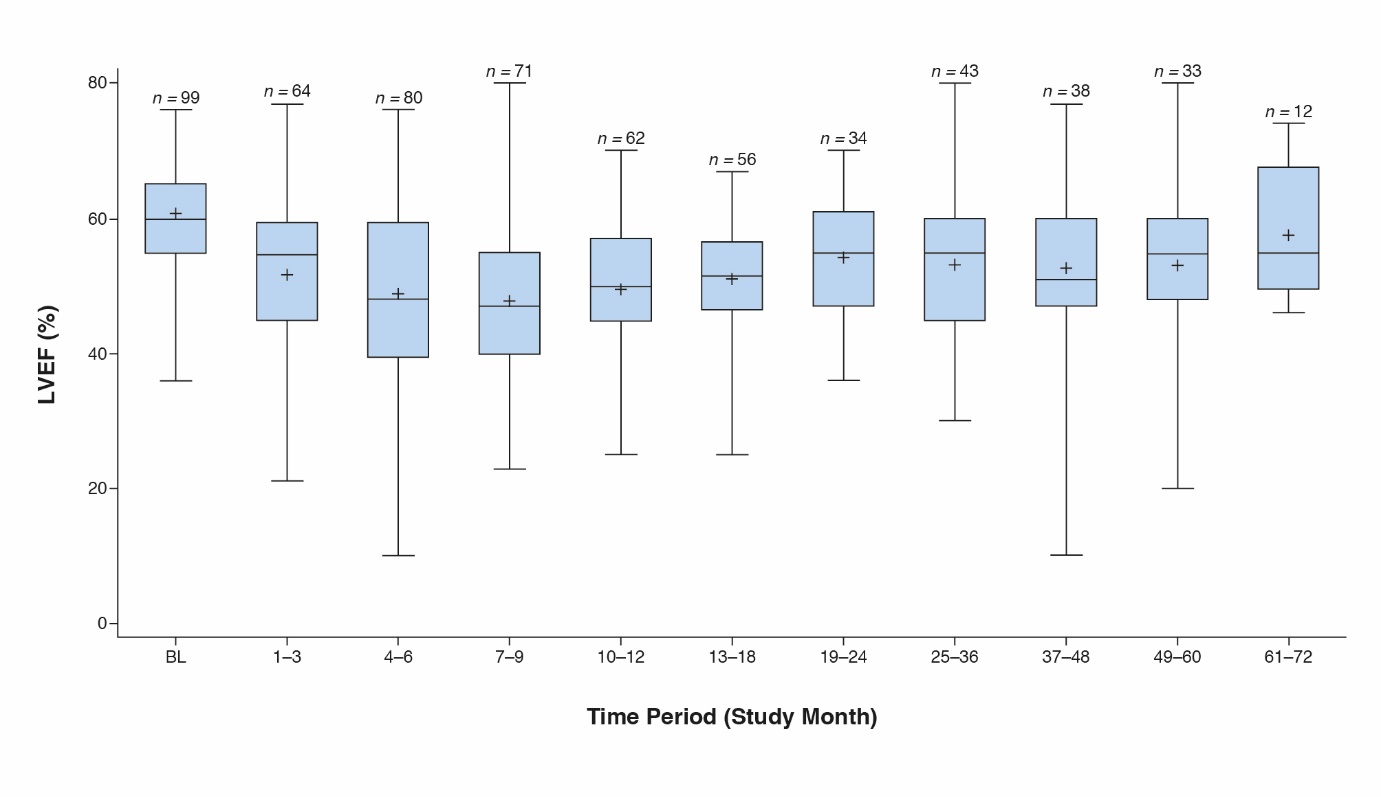

*BL* baseline, *LVEF* left ventricular ejection fraction
*N* is the number of patients with an LVEF assessment within the time period. In cases where a patient has multiple LVEF assessments within the same time period, the highest value for baseline and the lowest value in a time period was reported

**Supplementary Table 1** Concurrent therapies

Concurrent treatments were defined as all treatments which ended one day after the date of first trastuzumab administration or later, including any treatment given post-trastuzumab administration and prior to disease recurrence.

Data on concurrent therapies were not systematically collected in OHERA as at the time of study initiation, the approved indication of trastuzumab was for the treatment to be given as a monotherapy.

| **Concurrent therapy, *n* (%)** | **All patients**  **(*N* = 3733)** |
| --- | --- |
| Anticancer therapy (excl. radiotherapy and hormonal therapy) | |
| Patients who received at least one anticancer therapy | 783 (21) |
| Taxanes | |
| Patients with at least one treatment | 754 (20) |
| Docetaxel | 552 (15) |
| Paclitaxel | 212 (6) |
| Platinum compounds | |
| Patients with at least one treatment | 74 (2) |
| Carboplatin | 66 (2) |
| Cisplatin | 8 (< 1) |
| Alkylating agents | |
| Patients with at least one treatment | 66 (2) |
| Cyclophosphamide | 66 (2) |
| Cytotoxic antibiotics | |
| Patients with at least one treatment | 53 (1) |
| Epirubicin | 39 (1) |
| Doxorubicin | 14 (< 1) |
| Antimetabolites | |
| Patients with at least one treatment | 30 (< 1) |
| Fluorouracil | 28 (< 1) |
| Methotrexate | 6 (< 1) |
| 5-HT3 antagonists | |
| Patients with at least one treatment | 4 (< 1) |
| Granisetron | 4 (< 1) |
| Corticosteroids | |
| Patients with at least one treatment | 3 (< 1) |
| Dexamethasone | 3 (< 1) |
| Vinca alkaloids | |
| Patients with at least one treatment | 2 (< 1) |
| Vinorelbine | 2 (< 1) |
| Antiemetics | |
| Patients with at least one treatment | 1 (< 1) |
| Aprepitant | 1 (< 1) |
| Colony-stimulating factors | |
| Patients with at least one treatment | 1 (< 1) |
| Filgrastim | 1 (< 1) |
| Hormonal therapy | |
| Patients who received at least one hormonal therapy | 1859 (50) |
| Aromatase inhibitors | |
| Patients with at least one treatment | 1060 (28) |
| Letrozole | 505 (14) |
| Anastrozole | 490 (13) |
| Exemestane | 86 (2) |
| Aromatase inhibitor not otherwise specified | 21 (< 1) |
| Antiestrogens | |
| Patients with at least one treatment | 894 (24) |
| Tamoxifen | 894 (24) |
| Gonadotrophin and analogs | |
| Patients with at least one treatment | 217 (6) |
| Triptorelin | 79 (2) |
| Goserelin | 60 (2) |
| Leuprorelin | 49 (1) |
| Gonadotrophin-releasing hormone | 14 (< 1) |
| Gonadotrophin-releasing hormone analog | 14 (< 1) |
| LHRH agonist | 1 (< 1) |
| Radiotherapy | |
| Patients who received at least one radiotherapy treatment | 1115 (30) |
| Radiotherapy to breast | 801 (21) |
| Radiotherapy | 403 (11) |
| Radiotherapy to lymph nodes | 43 (1) |
| Brachytherapy | 5 (< 1) |
| Brachytherapy to breast | 2 (< 1) |

*LHRH* luteinizing hormone-releasing hormone

**Supplementary Table 2** Comparison of OHERA with other clinical trials

|  | **OHERA^a^** | **HERA  (H IV 1-year arm)^b^** | | | | **NSABP B-31**  **(AC→PTX + H IV arm)** | | | **NCCTG-N9831 (Alliance)**  **(AC→PTX→H IV [arm B] or AC→PTX + H IV → H IV [arm C)** | **BCIRG-006**  **(AC-T +  H IV arm)** | **BCIRG-006**  **(TC +  H IV arm)** |
| --- | --- | --- | --- | --- | --- | --- | --- | --- | --- | --- | --- |
|  |  | Piccart-Gebhart, 2005 [1] | Procter, 2010 [9] | Goldhirsch, 2013 [6] | Tan-Chiu, 2005 [13] | | Perez, 2011 [8] | Romond, 2012 [12] | Advani, 2016 [10] | Slamon, 2011 [3] | Slamon, 2011 [3] |
| Sample size | *N* = 3733 | *N* = 1677 | *N* = 1682 | *N* = 1682 | *N* = 850 | | Not reported | *N* = 947 | *n*= 710 [B]  *n*= 570 [C] | *N* = 1074 | *N* = 1075 |
| Median follow-up | 5 years | 1 year | 3.6 years | 8 years | 27 months | | 4 years | 7 years | 9.2 years | 5 years | 5 years |
| Symptomatic CHF (NYHA class II–IV) | 2.8% | 1.7%^c^ | 1.9% | Not reported | Not reported | | Not reported | Not reported | 2.7%  (19/710 [B])  3.5%  (20/570 [C]) | Not reported | Not reported |
| Severe CHF (NYHA class III/IV) | 1.0% | 0.5% (0.6%) | 0.8% | 0.8%^d^ | 3.6%^d^ | | 3.8%^d^ | 3.8% (36/947) | Not reported | 2.0% | 0.4% |
| Significant LVEF drop^e^ | 7.6%^f^ | 7.1% | 9.8%  (3.6% confirmed) | 4.1%  (confirmed) | 34%^g^ | | Not reported | Not reported | 35.3% [B]^h^  39.8% [C]^h^ | 18.6%^h^ | 9.4%^h^ |
| Cardiac deaths | 0.2% | 0 | 0 | Not reported | 0 | | Not reported | 0.1% (1/947) | 0.1% (1/710 [B]) 0.2% (1/570 [C]) | 0 | 0 |

*AC-T* doxorubicin and cyclophosphamide followed by docetaxel, *BCIRG* Breast Cancer International Research Group, *CHF* congestive heart failure, *H IV* intravenous trastuzumab, *LVEF* left ventricular ejection fraction, *NCCTG* North Central Cancer Treatment Group, *NSABP* National Surgical Adjuvant Breast and Bowel Project, *NYHA* New York Heart Association, *PTX* paclitaxel, *TC* docetaxel and carboplatin

Data are presented as % or *n*/*N*, as reported in the respective publications

^a^Excluding any events or assessments after recurrence of disease

^b^HERA study: a cardiac advisory board of three independent cardiologists was responsible for the review cases of CHF to determine whether such events satisfied the criteria regarding primary or secondary cardiac endpoints

^c^As defined by a cardiologist and significant decrease in LVEF

^d^Combined incidence of severe CHF (Grades 3/4) and cardiac death (although no cardiac death was reported)

^e^LVEF values based on the following patient populations: OHERA, *n* = 3291; Piccart-Gebhart, *n* = 1595; Goldhirsch, *n* = 1682. In HERA, a significant LVEF drop was defined as an absolute decline of ≥ 10 percentage points from baseline LVEF and to < 50% [9]

^f^Incidence of LVEF drop in OHERA was evaluated in patients treated with H IV who had one numerical baseline LVEF value and at least one numerical post-baseline LVEF value

^g^For 1 year of follow-up; includes declines in LVEF by ≥ 10 percentage points from baseline and to < 55% (rather than < 50%)

^h^Defined as LVEF drop ≥ 10%; data are based on *n* = 710 and *n* = 570 patients in arms B and C of NCCTG-N9831 (Alliance), and *n* = 1042 for the AC-T plus H IV arm and *n* = 1031 for the TC plus H IV arm of BCIRG-006
